# Ultrasound Assisted Green Synthesis of Silver and Iron Oxide Nanoparticles Using Fenugreek Seed Extract and Their Enhanced Antibacterial and Antioxidant Activities

**DOI:** 10.1155/2019/1714358

**Published:** 2019-04-08

**Authors:** Aarti R. Deshmukh, Arvind Gupta, Beom Soo Kim

**Affiliations:** Department of Chemical Engineering, Chungbuk National University, Cheongju, Chungbuk 28644, Republic of Korea

## Abstract

This study reports a facile and ecofriendly approach for the ultrasound assisted synthesis of silver and iron oxide nanoparticles and their enhanced antibacterial and antioxidant activities. The fenugreek seed extract was used as reducing, capping, and stabilizing agent in the synthesis of nanoparticles. The transmission electron microscopy results showed that nanoparticles synthesized by ultrasonication have a smaller size (~20 nm) as compared to the nanoparticles fabricated by magnetic stirring (~40 nm). The color change of the solution from milky white to brown suggested the formation of silver nanoparticles which was confirmed by the presence of an absorbance peak at 396 nm. The results of powder X-ray diffraction and energy dispersive X-ray spectroscopy confirmed the crystallinity and elements present in nanoparticles synthesized using fenugreek seed extract. Fourier transform infrared analysis showed that the fenugreek seed phytochemicals were coated on the nanoparticle surface. Thermal gravimetric analysis showed the thermal degradation and stability of nanoparticles. Magnetization study of iron oxide nanoparticles confirmed the superparamagnetic nature. The silver nanoparticles showed antibacterial activities against both gram-negative (*Escherichia coli*) and gram-positive (*Staphylococcus aureus*) bacteria, while no antibacterial activities were observed for iron oxide nanoparticles. The ultrasound assisted nanoparticles showed higher stability and antibacterial and antioxidant activity compared with the nanoparticles fabricated by magnetic stirring.

## 1. Introduction

The emerging unique properties and applications of nanoparticles have made nanotechnology one of the most interesting areas of research. The size, shape, and morphology are known to govern the properties of nanoparticles and many studies have been reported with a special emphasis on controlling the size and shape of nanoparticles. Though there are many methods of synthesizing nanoparticles such as chemical and physical method, green synthesis of nanoparticles has gained prominence in recent years [1, 2]. The green synthesis involves the usage of various reducing agents such as plant extracts and microbes [3–5]. However, the plant extract synthesized nanoparticles have acquired significant importance due to their cost effectiveness, stability, ease of availability, and preparation, compared to the nanoparticles synthesized by chemicals and microbes. Plant extracts such as gallnut extract [6],* Kalopanax pictus* extract [7], etc. have been explored in order to synthesize metal based nanoparticles i.e., silver, gold, iron oxide, indium oxide, copper, palladium, selenium nanoparticles, manganese dioxide, etc.

Fenugreek seed extract is an herb which belongs to the Leguminosae family, broadly found in the Indian continent and can be used for the synthesis of nanoparticles. The fenugreek seed contains diosgenin, tigogenin, gitogenin, and saponin (fenugrin B) along with several alkaloids. The major bioactive compounds in fenugreek seed are believed to be polyphenolic compounds such as isovitexin and rhaponticin [8, 9]. The chemical composition of the fenugreek endosperm shows the relatively higher protein (43.8 g/100 g) and saponin (4.6 g/100 g) content. Additionally, seed husk is found to be rich in total polyphenols. Fenugreek seed extract also exhibits several pharmacological properties such as antiviral, anti-inflammatory, antitumor, antioxidant activity, etc. [8, 10].

Silver has been used in medicine since a very long time [5]. Additionally, silver nanoparticles are known to have antibacterial, anticancer, antifungal, and enzyme mimicking activity [11–17]. Iron oxide magnetic nanoparticles have been used in various fields such as targeted drug delivery, magnetic resonance imaging, cell separation and sorting in magnetic hyperthermia, biosensors, nanocomposites, and microfluidic paper devices for the detection of heavy metals [18–21]. Magnetic nanoparticles used for the diagnosis should be free of agglomerates, be super-magnetic at room temperature, and be biocompatible and nontoxic. [22]. Therefore, it is necessary to enhance the synthesis method to improve the physicochemical properties of magnetic nanoparticles. The properties of nanoparticles are affected by the method of preparation, experimental conditions, and interactions of metal ions with reducing agents. The size, shape, and distribution of nanoparticles can be controlled by changing the method of synthesis and the types of reducing and stabilizing agents [23, 24].

Ultrasound assisted synthesis of various types of nanoparticles and nanostructured materials containing transition metals, carbon materials, and noble metals have received significant attention in recent years [25]. The key factor involved in the ultrasonic assisted processes is cavitation wherein there is a formation, growth, and collapsing of bubbles with high pressure, high local temperature and energy in the liquid medium. The formation of bubbles due to the cavitation during ultrasonication leads to higher yield with nonaggregated nanoparticles. Hence, the ultrasound assisted method is considered to be an effective method for the synthesis of nanoparticles [26–32].

The current study deals with the ultrasound assisted green synthesis of silver and magnetic iron oxide nanoparticles using fenugreek seed extract and their characterization along with exploring their antibacterial and antioxidant activities for targeted applications. In addition, the effect of ultrasonication on morphology and properties of silver and iron oxide nanoparticles are discussed. The result proved that the ultrasound assisted synthesis process improves the stability of nanoparticles. Although many studies have reported the synthesis of various nanoparticles using plant extracts, ultrasound assisted green synthesis of silver and iron oxide nanoparticles is very rare among them. To the best of our knowledge, this is the first comparative study addressing the sonication effect on the green synthesis of nanoparticles and their enhanced antibacterial and antioxidant activities.

## 2. Experimental Methods

### 2.1. Reagents

Silver nitrate (analytical grade, 99.0%) was purchased from Jinchemical Co., Ltd. (Korea). Iron (III) chloride hexahydrate (FeCl_3_·6H_2_O, 99.0%) and iron (II) chloride tetrahydrate (FeCl_2_·4H_2_O, 97.0%) were purchased from Junsei Chemical Co. Ltd. (Japan). 2,2-Diphenyl-1-picrylhydrazyl (DPPH) was purchased from Sigma-Aldrich. Sodium borohydride (99.6%) was purchased from TCI Japan. 28% ammonia solution was purchased from OCI Company Ltd. (Korea). Fenugreek seeds (1% moisture content) were procured from local market in India. All chemicals were used as received, without further purification. For the preparation of all solutions, distilled water was used.

### 2.2. Bacterial Culture

The bacterial strains* Staphylococcus aureus *(ATCC 6538) and* Escherichia coli *(ATCC 11775) were used for determination of antibacterial activity. Nutrient agar medium was used to culture the strains at 37°C and preserved at -80°C in glycerol stock vials for further study.

### 2.3. Extract Preparation from Fenugreek Seed

Fenugreek seed was washed thoroughly with distilled water to remove the dust particles followed by drying in oven at 80°C for 1 h. The seeds were ground with mortar and pestle and stored in glass bottles. The ground seed powder (5 g) was boiled in 100 ml distilled water under reflux condition at 100°C for 1 h. Then, it was filtered using 0.2 *μ*m filter paper and the broth as extract was stored at 4°C and used within two weeks. The yield for the extract was found to be 1.43±0.1 g/5 g (28.6 wt%) of fenugreek seed.

### 2.4. Synthesis of Nanoparticles Using Fenugreek Seed Extract

#### 2.4.1. Ultrasound Assisted Fabrication of Silver Nanoparticles

**Figure 1:**
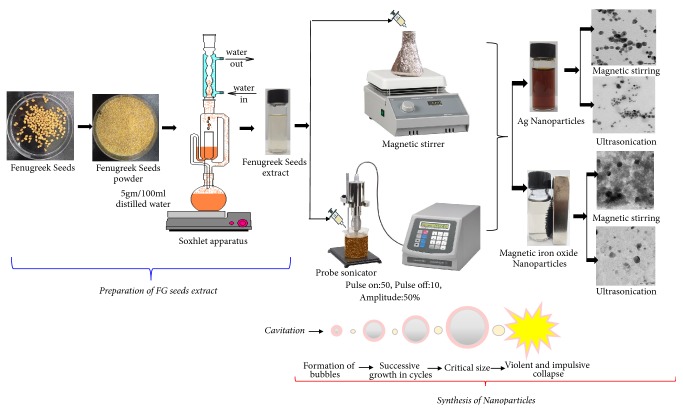
Scheme for the synthesis of nanoparticles using fenugreek seed extract.

The silver nanoparticles were prepared using fenugreek seed extract as a reducing and capping agent. The scheme for the synthesis of nanoparticles is shown in Figure 1. In a typical experiment, 5 ml of fenugreek seed extract (3.28 mg/ml) was added dropwise into 50 ml of 1 mM silver nitrate solution in a conical flask under continuous stirring at room temperature at 500 rpm for 1 h. To avoid photo-degradation, flask was covered with aluminum foil. The same procedure was followed for ultrasound assisted synthesis with a probe sonicator (Qsonica, pulse on: 50, pulse off: 10, amplitude: 50%). During the ultrasound assisted synthesis, sonication was applied for 15 min followed by stirring at 400 rpm for 15 min. As the reaction proceeded, the color of the mixture was changed from colorless to pale yellow to brown, which suggested the formation of silver nanoparticles. Finally, the obtained silver nanoparticles were centrifuged at 15000 rpm for 20 min followed by lyophilization. The samples were named as AgM and AgU for silver nanoparticles formed by stirring and ultrasound, respectively.

#### 2.4.2. Ultrasound Assisted Synthesis of Iron Oxide Nanoparticles

Iron oxide nanoparticles were synthesized by the coprecipitation method [33] with slight modification. Initially, 50 ml of fenugreek seed extract was added to 100 ml of 1 M FeCl_2_ and 2 M FeCl_3_, followed by stirring at room temperature for 2 h at 400 rpm. Later, 10 ml of 25% ammonia solution was added to the suspension under continuous stirring for 1 h. The nanoparticles were separated by magnet and dried in hot air oven at 60°C. For the ultrasound assisted synthesis, 10 ml of 28% aqueous ammonia solution was added dropwise into the suspension under ultrasonication with a probe sonicator (Qsonica, pulse on: 50, pulse off: 10, amplitude: 50%) for 15 min followed by stirring at 400 rpm for 15 min. Then, formed magnetic iron oxide nanoparticles were separated by magnetic bar and dried in hot air oven at 60°C. The samples were named as FeM and FeU for iron oxide nanoparticles formed by stirring and ultrasound, respectively.

#### 2.4.3. Chemical Synthesis of Silver and Iron Nanoparticles

Silver nanoparticles were prepared by chemical reduction method using sodium borohydride as a reducing agent [34, 35]. In typical experiment, 50 ml of 1 mM silver nitrate solution was kept in aluminum foiled cover conical flask and 5 ml of 0.1 M sodium borohydride was added dropwise. Solution was mixed vigorously at 400 rpm for 1 h at room temperature. The obtained colloidal suspension was used for the stability study.

Iron oxide nanoparticles were obtained by chemical method at room temperature using ammonia solution without fenugreek seed extract [36]. Initially, 100 ml of 1M FeCl_2_ and 2M FeCl_3_ was mixed together. 10 ml of 28% aqueous ammonia solution was added dropwise followed by stirring at room temperature for 2 h. The obtained colloidal suspension was used for the stability study.

### 2.5. Characterization of Nanoparticles

#### 2.5.1. UV-Visible Spectrophotometry (UV-Vis)

Formation of nanoparticles was monitored by using UV-visible spectrophotometer. The spectrum was obtained on UV mini-1240 (Shimadzu) operated at a resolution of 10 nm in 200-800 nm wavelength range.

#### 2.5.2. Fourier Transform Infrared Spectroscopy (FTIR)

The synthesized nanoparticles were subjected to Fourier transform infrared (FTIR) analysis to determine the capping of biomolecules on nanoparticles. The FTIR analyses of fenugreek seed, silver nitrate, silver nanoparticles, iron (II) chloride tetrahydrate, iron (III) chloride hexahydrate, and iron oxide nanoparticles were obtained on IR200 FTIR spectrometer (Thermo Scientific) in the wavenumber range 400-4500 cm^−1^ with a resolution of 4 cm^−1^ after 32 scans.

#### 2.5.3. Scanning Electron Microscopy and Energy Dispersive X-Ray Spectroscopy (SEM-EDX)

The morphology and elemental composition of the synthesized nanoparticles were analyzed with scanning electron microscope (SEM, LEO-1530) equipped with energy dispersive X-ray spectroscope (EDX) instrument. The sample was prepared by dropping a small amount of dried nanoparticles on carbon tape.

#### 2.5.4. Powder X-Ray Diffraction (XRD)

The composition and crystalline structure of nanoparticles were characterized by using high resolution X-ray diffractometer (XRD, X'Pert-Pro, PANalytical) operated at room temperature with a voltage of 40 kV, with CuK*α* radiation at *λ*=1.5406 Å. The samples were scanned in the 2*θ* range of 10° to 100° with the rate of 2°/min.

#### 2.5.5. Transmission Electron Microscopy (TEM)

Transmission electron microscopy (TEM, Libra 120, Carl Zeiss) analyses of samples were carried out to obtain a quantitative measure of particles, size distribution, and morphology. The TEM samples were obtained by dropping colloidal nanoparticle solution on the carbon coated copper grid and evaporating the solvent at room temperature.

#### 2.5.6. Thermogravimetric Analysis

TGA analyzer (TGA6, Perkin Elmer) was used to record the thermograms in the temperature range from 25°C to 900°C with a heating rate of 10°C/min under nitrogen atmosphere.

#### 2.5.7. Magnetic Measurements

The magnetic performance was assessed using a magnetic property measurement system (MPMS3-Evercool, Quantum Design Inc) operated at 300 K. Samples were scanned in the range of -36000 to 36000 Oe of magnetic field.

### 2.6. Antibacterial Activity Assay

The agar diffusion method was used for the antimicrobial assay. The appropriate solidified medium was inoculated with 100 *μ*l of bacterial inoculum and spread over the plates using a sterile bud in order to get a uniform microbial growth plate. Sterile paper discs (10 mm diameter, Advantec, Japan) were impregnated with 25, 50, 75, and 100 *μ*l of each fenugreek seed extract, nanoparticles synthesized by magnetic stirring and ultrasonication, and placed on the agar surface using forceps dipped in ethanol and flamed. To avoid contamination, all Petri dishes were sealed with parafilm tape. The dishes were incubated at 37°C for 24 h. After the incubation period, the inhibition zone was monitored and the diameter of the inhibition zone was calculated by visualizing the blank space around the paper disc to evaluate the antibacterial performance of the fenugreek seed extract and nanoparticles.

### 2.7. Antioxidant Activity Assay

Antioxidant activity assay was performed by evaluating radical scavenging activity of fenugreek seed extract and the synthesized nanoparticles. The radical scavenging activity of the fenugreek seed extract was determined by DPPH assay according to the method [37]. The decrease in the absorbance at 517 nm was measured in a 48-well plate containing 1 ml of 0.1 mM ethanolic DPPH solution (standard) mixed with varying concentrations of 10, 30, 50, 70, 90, and 110 *μ*g/ml of test samples. The plate was kept in the dark for 30 min, followed by measuring the absorbance in BIO-RAD xMark™ microplate absorbance spectrophotometer. The radical scavenging activity of all samples to scavenge DPPH radical was calculated by using

(1) DPPH  scavenge  Activity=1−Absorbance  of  SampleAbsorbance  of  Standard×100

## 3. Results and Discussion

### 3.1. Ultrasound Assisted Synthesis and Characterization of Nanoparticles

**Figure 2:**
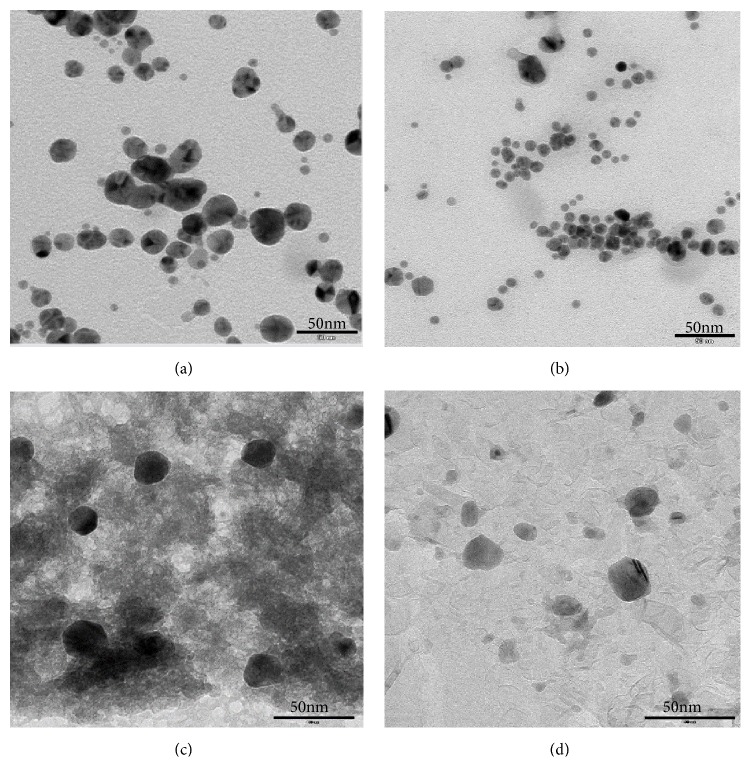
TEM images of (a) AgM, (b) AgU, (c) FeM, and (d) FeU.

The fabricated silver and iron oxide nanoparticles were characterized by TEM to analyze the size and morphology as shown in Figure 2. It was found that the size and shape of silver and iron oxide nanoparticles synthesized using fenugreek seed extract by magnetic stirring and ultrasonication were irregular in size and nearly spherical in shape. The average particle size of silver nanoparticles prepared by magnetic stirring was found to be approximately 40 nm, whereas the synthesis mediated with ultrasound produced lesser sized (~20 nm) silver nanoparticles. In case of iron oxide nanoparticles, ultrasound affected the size of the particles which was found to be ~20 nm in comparison to synthesis without using ultrasound (~35 nm). The microscopic analysis confirms the formation of silver and iron oxide nanoparticles which was found to be spherical in shape and ultrasound affected the fabrication process resulting in the reduction in size of the nanoparticles.

**Figure 3:**
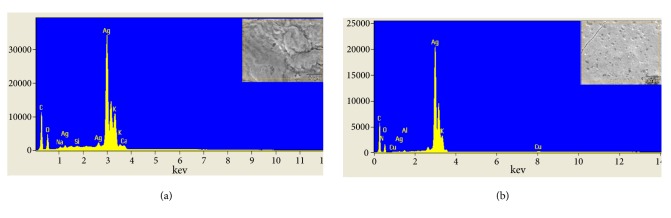
EDX spectra for (a) AgM and (b) AgU along with SEM image area (inset).

**Figure 4:**
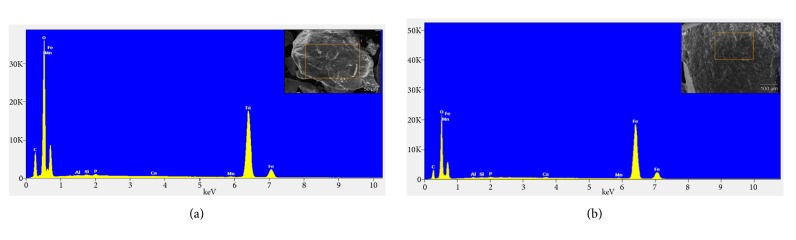
EDX spectrum for (a) FeM and (b) FeU along with SEM image area (inset).

EDX technique was employed to confirm the formation of silver and iron oxide nanoparticles. As shown in Figures 3(a) and 3(b), a strong signal at ~2.98 keV was found, which is the typical absorption signal of metallic silver, confirming the presence of elemental silver in both nanoparticles. This also supports the hypothesis that silver nitrate can be reduced by fenugreek seed extract. In addition, the signal for C, O, N, Si, Ca, and K illustrates the existence of biomolecules as stabilizing agent on the surface of silver nanoparticles. The atomic percent composition analysis of synthesized nanoparticles illustrates that there is more Ag^+^ reduction in sonication process as compared to magnetic stirring. The weight percent of Ag formed is 65.6% for magnetically stirred synthesis which is increased to 75.8% for ultrasound assisted synthesis. Similarly, EDX for iron oxide nanoparticle synthesis is shown in Figures 4(a) and 4(b). The weight percent of Fe formed is 52.5% and 66.4% for magnetic stirred and ultrasonic assisted nanoparticles, respectively, which confirms the presence of iron elements in the compounds. EDX spectra revealed the presence of iron peaks in three different areas at 0.7, 6.4, and 7.2 keV. For both silver and iron oxide nanoparticles, ultrasonication showed higher rate of reduction and yield.

The bioreduction of Ag^+^ was postulated as the trapping of Ag^+^ ions on the protein surface due to electrostatic interactions between Ag^+^ and proteins present in plant extract [38]. The possible chemical reaction for preparing Ag nanoparticles with fenugreek seed extract (FG) can be presented as

(2) $$\begin{array}{c} {Ag}^{+}+FG\to {Ag}^{o}/FG \end{array}$$

For magnetic iron oxide nanoparticles, the possible chemical reactions can be given as follows [19, 39]:

(3) 2Fe3++Fe2++FG+H2O→2Fe3+:Fe2+/FG

(4) 2Fe3+:Fe2+/FG+O2→Iron  oxide  nanoparticles/FG

**Figure 5:**
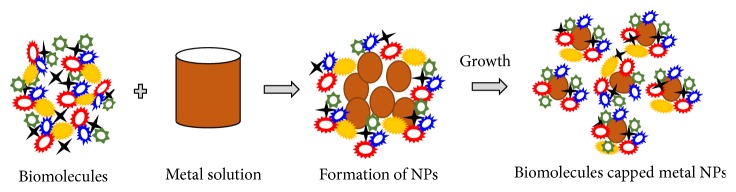
Schematic representation of possible mechanism for the biosynthesis of nanoparticles.

Several researchers have identified approximately 18 different phenolic compounds in the fenugreek seed extract [40]. These biomolecules, present in aqueous extract of fenugreek seed, bind on the surface of metals and could be involved in the stabilization of nanoparticles. The illustration for binding of biomolecules with metal molecules is shown in Figure 5.

**Figure 6:**
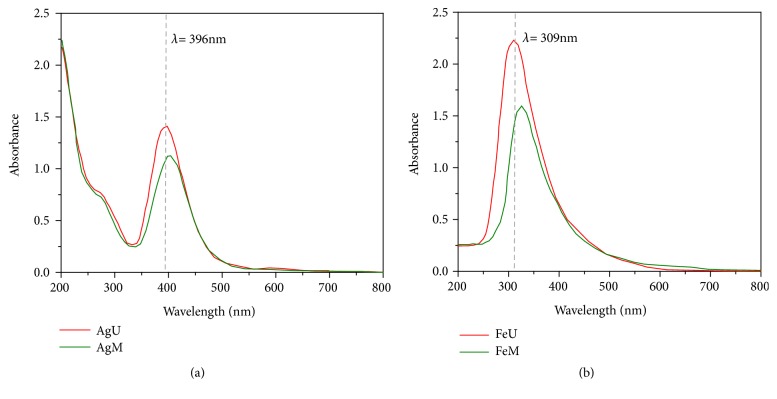
UV-Vis spectroscopy of (a) silver and (b) iron oxide nanoparticles.

UV-Vis analysis is useful in metallic nanoparticle characterization and can acquire certain information about shape, size, and stability of nanoparticles. The plasmonic absorbance phenomenon of nanoparticles makes it detectable by UV-Vis spectrophotometer.  Figure 6 shows the UV-Vis spectra for silver and iron oxide nanoparticles recorded from the colloidal medium after the experiment at room temperature. The absorption spanned a wide range from 340-560 nm with prominent peaks at 398 and 396 nm for AgM and AgU, respectively (Figure 6(a)). This peak indicates the formation of silver nanoparticles, as it is within the range of the surface plasmon resonance (SPR) for AgNPs. For iron oxide nanoparticles, the absorption spanned a wide range from 240-650 nm with prominent peaks at 335 and 309 nm for FeM and FeU, respectively (Figure 6(b)). The broad SPR peak that extends in the wide range with an absorption tail could be multisize distribution of nanoparticles. The wavelength is the function of particle size. It is observed that the SPR shifts towards the high wavelength as the diameter of nanoparticles increases, which is evident with the TEM analysis. It is plausible to say that the advantage of ultrasound assisted synthesis is to produce relatively smaller nanoparticles compared to the synthesis with magnetic stirring [41–43].

**Figure 7:**
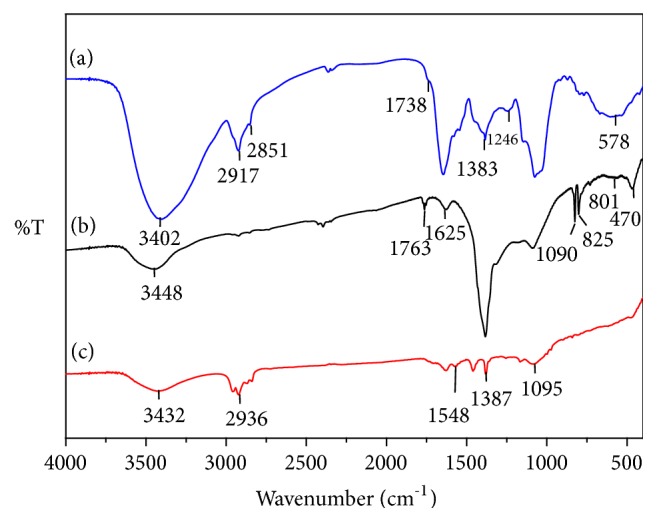
FTIR of (a) fenugreek seed extract, (b) silver nitrate, and (c) silver nanoparticles.

As a powerful tool, FTIR was used for the analysis of the functional molecular vibrations. The FTIR spectrum of the fenugreek seed extract, silver nitrate, and silver nanoparticles are shown in Figure 7. The peaks for fenugreek seed extract shown in Figure 7(a) at 3368, 2927, 1743, 1648, 1543, 1384, 1242, 1073, and 586 cm^−1^ correspond to N-H as well as OH stretching vibration from protein, stretching vibration of asymmetric and symmetric C-H, C=O stretching, C=N of protein secondary amine bending vibration, C-C, C-H bending, CH-OH stretching, Si-O-Si stretching, and Si-H stretching. The functional groups responsible for the formation of the silver nanoparticles are identified by comparing the functional groups present in the three samples. The peaks for silver nitrate in Figure 7(b) at 2394, 1759, 1627, 1406, and 823 cm^−1^ represent C-H, C=O, C=C stretching vibrations and C-H bending, respectively. The silver nanoparticles synthesized from fenugreek seed extract are shown in Figure 7(c). The peaks at 1630, 1459-1380, and 1183 cm^−1^ represent the C=C stretching, C-H bending, and C-N bending, respectively [44]. The peak at 1380 cm^−1^ represents the silver ions binding the polysaccharides [45]. The presence of the broad peak around 3411 cm^−1^ in both fenugreek seed and silver nanoparticles can be due to the presence of organic moiety like carboxylic acid functional group and O-H stretching vibration along with N-H stretching vibration. FTIR studies reveal that amino acids, peptides, and proteins can bind with metal, cap the particles, and stabilize the silver nanoparticles against agglomeration. This demonstrates that saponin, proteins, and polyphenols from fenugreek seed extract can be involved in the formation of silver nanoparticles.

**Figure 8:**
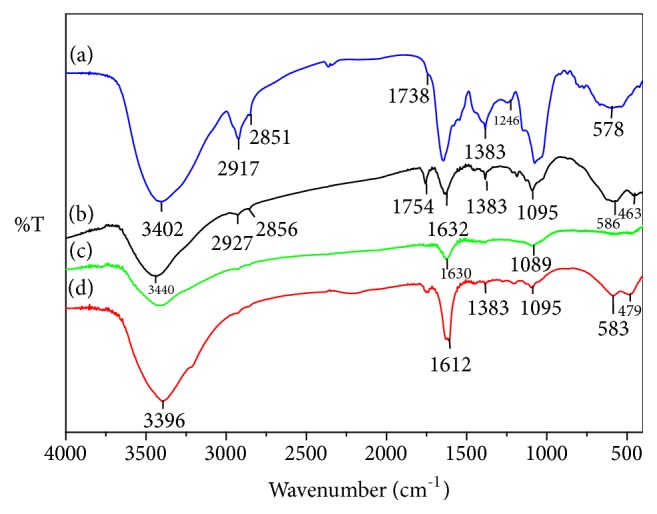
FTIR of (a) fenugreek seed extract, (b) iron oxide nanoparticles, (c) iron (II) tetrahydrate, and (d) iron (III) hexahydrate.

As in Figure 8(b), iron oxide nanoparticles showed a broad peak at 3440 cm^−1^, indicating the presence of N-H as well as O-H stretching. The band at 2927 cm^−1^ implies the stretching vibration of symmetric and asymmetric C-H. The absorption band at 1632 cm^−1^ can be attributed to the presence of carbonyl stretching and that at 1400 cm^−1^ can be due to C-N stretching or O-H bending vibrations [46]. However, the band at 1095 cm^−1^ can be assigned to C-OH vibrations attributed to proteins present in fenugreek seed. The presence of iron oxide nanoparticles can be confirmed by the strong absorption band at 586 cm^−1^ [47, 48].

**Figure 9:**
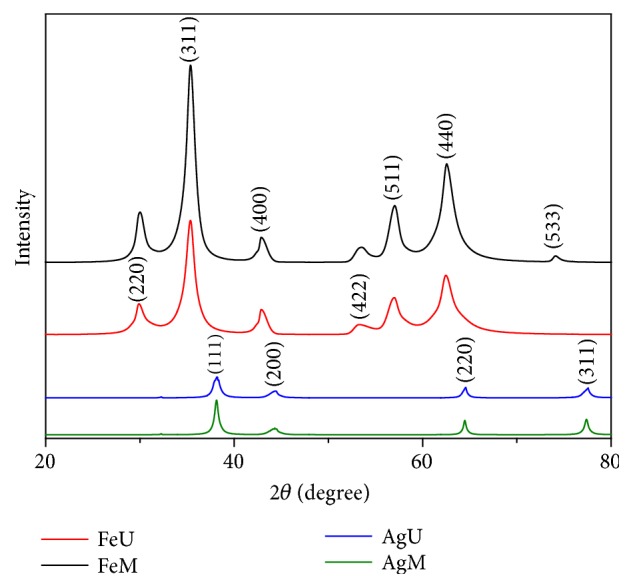
XRD pattern of silver and iron oxide nanoparticles.

The crystalline nature of the obtained nanoparticles was further determined by XRD analysis. The typical XRD patterns of the fenugreek seed extract capped silver and iron oxide nanoparticles are shown in Figure 9. From the XRD spectra of AgM and AgU, distinct peaks at 38.1, 44.4, 64.4,77.3, and 81.4° are indexed as (1 1 1), (2 0 0), (2 2 0), and (3 1 1) plane of the face centered cubic metallic silver having d values of 2.358, 2.037, 1.443, 12.23, and 1.095, respectively. Amongst all Bragg's reflection, the (1 1 1) reflection is more intense, which indicates that silver nanoparticles are (1 1 1) oriented and highly anisotropic. The planes are in congruence with those reported in earlier studies [11, 37].

XRD distinct peaks of iron oxide nanoparticles synthesized by magnetic stirring (FeM) and ultrasonication (FeU) are indexed at the 2*θ* values of 29.8, 35.3, 42.8, 53.2, 57.0, and 62.4° representing (2 2 0), (3 1 1), (4 0 0), (4 2 2), (5 1 1), and (4 4 0) crystallographic planes, respectively. Their presence represents the cubic structure of magnetic iron oxide nanoparticles [36, 49–52]. The sharp peaks indicate the highly crystalline nature of the synthesized nanoparticles. The average crystallite size D for the nanoparticles was estimated from the diffractogram using the Debye Scherrer equation (5),

(5) $$\begin{array}{c} D=\frac{k\lambda }{\beta \times \cos\theta } \end{array}$$

where k, *λ*, *β*, and *θ* represent the shape dependent Scherrer's constant, wavelength of the X-ray used in the diffraction, full width at half maximum (FWHM) of a peak, and the diffraction angle, respectively [53]. The calculated average crystallite sizes of nanoparticles for four samples are given in Table 1.

**Figure 10:**
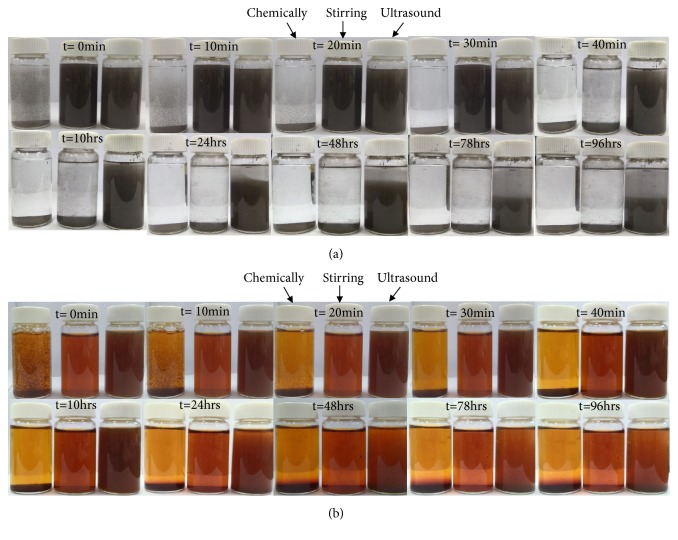
Demonstration of the stability of chemically synthesized, fenugreek seed extract magnetically stirred, and ultrasound assisted synthesized (a) silver and (b) iron oxide nanoparticles.

The suspension stability of the synthesized nanoparticles was analyzed using visual inspection of the dispersion solution of nanoparticles in water. 20 ml of each chemical and fenugreek seed extract nanoparticles were transferred to glass bottles and allowed to settle as shown in Figure 10. The stability behaviour was observed till 96 h. It was found that the chemically synthesized silver and iron oxide nanoparticles were less stable which tend to settle down from the beginning. Moreover, it was found that silver and iron oxide nanoparticle suspensions synthesized using ultrasonication process were stable till 10 h in comparison to nanoparticles synthesized by stirring (30 min). As discussed in the previous sections, the nanoparticles tend to be functionalized with the compounds present in the fenugreek seed extract and can be responsible for the stabilization of the nanoparticles in water.

**Figure 11:**
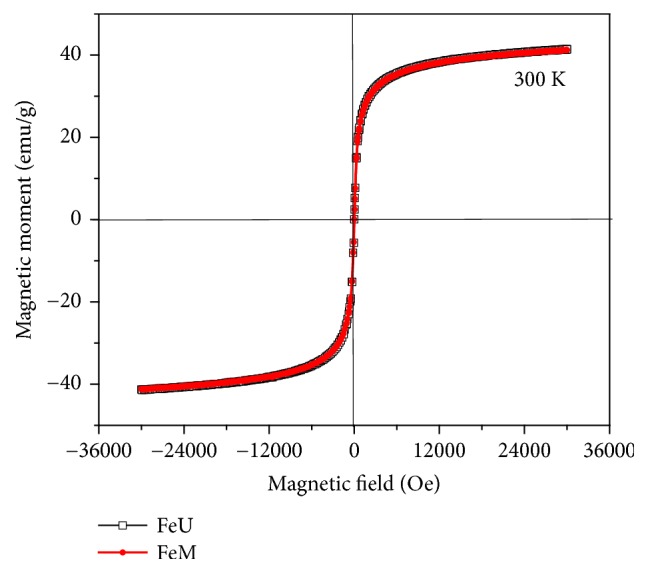
Magnetic moment versus applied magnetic field at 300 K.

Figure 11 demonstrates the magnetization curves of the iron oxide nanoparticles prepared with magnetic stirring and ultrasonication at 300 K. The magnetization curve for both nanoparticles exhibits no hysteresis, representing the superparamagnetic characteristics [54]. Superparamagnetic characteristic is extremely important for several applications such as magnetic resonance imaging, drug delivery, to heat tumor cells in an alternating magnetic field, etc. The magnetic material should not retain any magnetism after the magnetic field is removed. Saturation magnetization of the synthesized nanoparticles was found to be ~41.2 emu/g.

**Figure 12:**
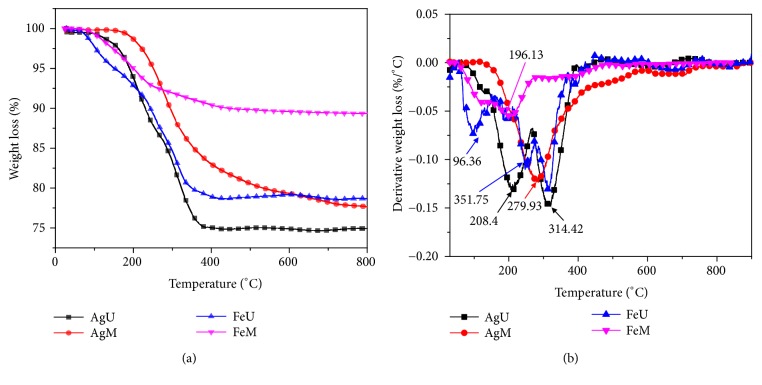
(a) TGA and (b) their derivative curves of the prepared nanoparticles.

TGA was conducted to understand the thermal degradation and thermal stability of the nanoparticles and fenugreek biomolecules present on the surface of nanoparticles. The thermograms are given in Figure 12. All samples showed two successive weight losses in the temperature region of 25°C-900°C. The initial weight loss was attributed to the evaporation of bound water molecules [55]. Other weight loss was found to be consequence of thermal degradation of biomolecules, which may present on the surface of nanoparticles as a stabilizing and capping agent. Overall, the total weight loss of 10-26% was observed. All the prepared nanoparticles showed high thermal stability. In case of the nanoparticles synthesized by ultrasonication, the percent weight loss was found to be higher in comparison to the magnetic stirred synthesis. As discussed in TEM and EDX analysis, the size of nanoparticles was smaller in ultrasonically synthesized nanoparticles which ultimately results in increased surface area; therefore there are more biomolecule moieties on the surface compared to the nanoparticles synthesized using magnetic stirring.

### 3.2. In Vitro Antibacterial Activity

**Figure 13:**
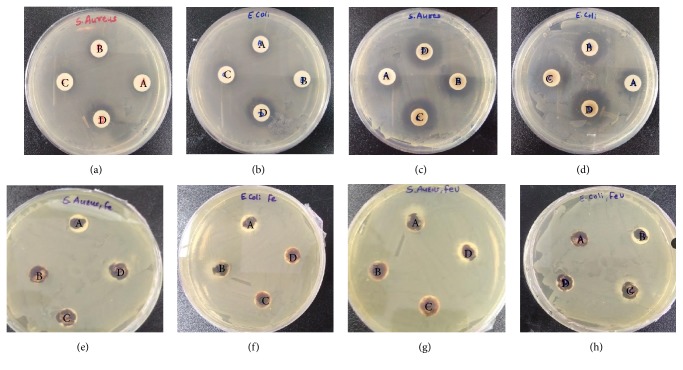
Petri plates showing the zone of inhibition of AgM on (a)* S. aureus* and (b)* E. coli*, AgU on (c)* S. aureus* and (d)* E. coli*, FeM on (e)* S. aureus* and (f)* E. coli*, and FeU on (g)* S. aureus* and (h)* E. coli* (A: 25 *μ*L, B: 50 *μ*L, C: 75 *μ*L, and D: 100 *μ*L of colloidal silver solution).

The results of the antibacterial activity of the silver and iron oxide nanoparticles synthesized by means of magnetic stirring and ultrasound assisted method against two bacteria* S. aureus *and* E. coli* are shown in Figure 13. The main principle behind the antibacterial activity of silver nanoparticles is that these particles create oxidative stress by generating reactive oxygen species inside the cell membrane and cause the disruption of cell membrane. It is known that silver nanoparticles exhibit antibacterial activity against both gram-positive and gram-negative bacteria. Depending on the extent of the membrane destruction, silver nanoparticles can reach the cytoplasm, interconnect with sulfur-containing proteins and enzymes, and interfere with the replication of DNA [56–58]. From the analysis, it was found that the ultrasound assisted synthesized nanoparticles showed more antibacterial activity than nanoparticles synthesized using magnetic stirring. As discussed in TEM micrograph, the size of nanoparticles was smaller in ultrasonically synthesized nanoparticles which ultimately results in increased surface area and therefore nanoparticle surface containing comparatively higher amount of biomolecule moieties. TGA also reveals that there are approximately 26 wt% of biomolecule moieties adhering to the nanoparticles. Therefore, it can be concluded that the antibacterial activity is the synergistic effect of particle size and the capped biomolecules [37, 56].

**Figure 14:**
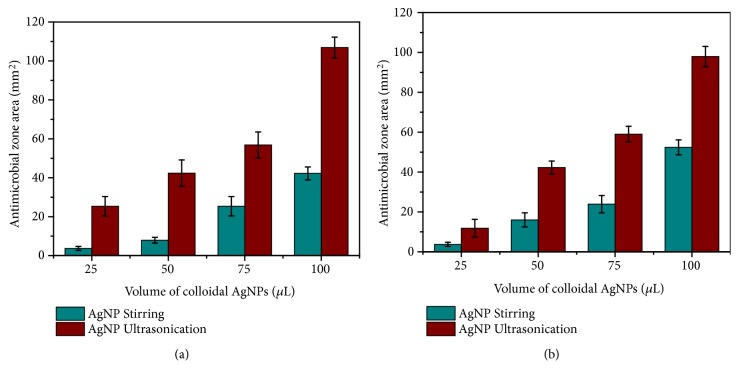
Antibacterial activity of silver nanoparticles against (a)* E. coli *and (b)* S. aureus.*

Compared to silver nanoparticles, iron oxide nanoparticles were found to be ineffective in antibacterial activity against* S. aureus* and* E. coli* (Figures 13(e)–13(h)). It is observed from graphical representation (Figures 14(a) and 14(b)) that the area of inhibition increases with increasing the concentration of nanoparticles.

### 3.3. Antioxidant Assay

**Figure 15:**
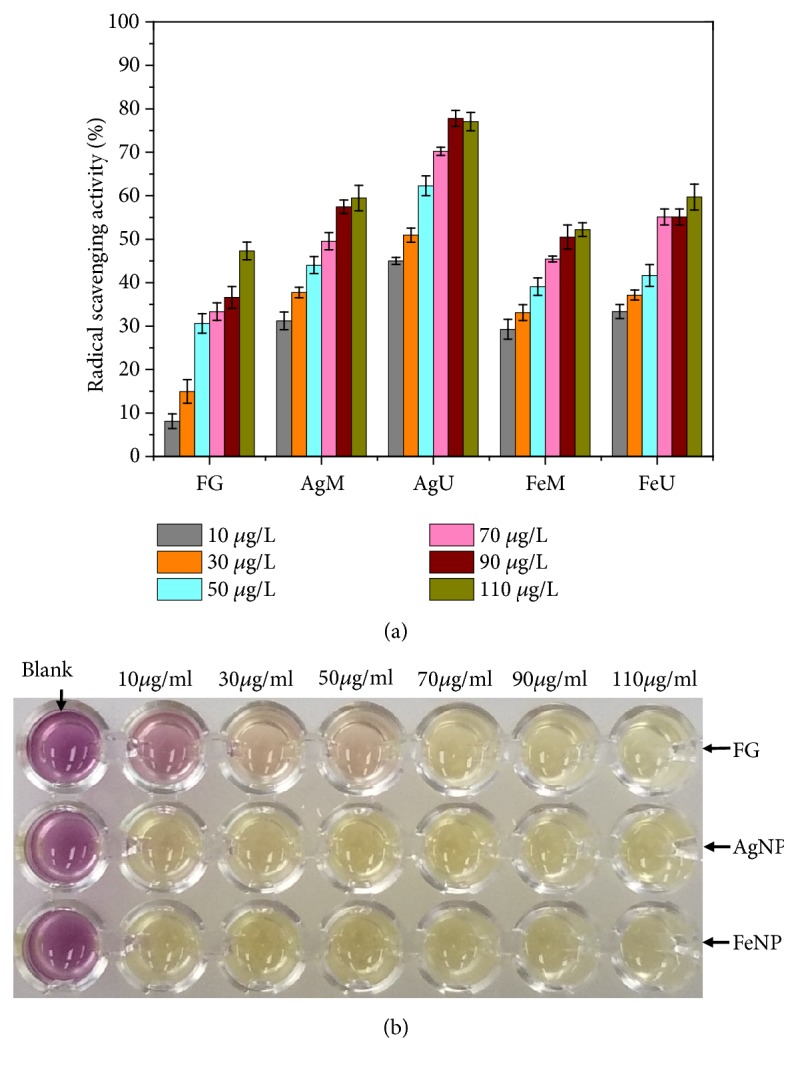
(a) DPPH assay of the synthesized silver and iron oxide nanoparticles and fenugreek seed extract; (b) color change due to radical scavenging.

The dose-dependent radical scavenging activity of fenugreek seed extract and the synthesized nanoparticles are shown in Figure 15. It can be observed that upon increasing the concentration of fenugreek seed extract and nanoparticles, reducing power increases. The maximum radical scavenging activity for fenugreek seed extract, AgM, AgU, FeM, and FeU nanoparticles found at 110 *μ*g/ml was 48, 60, 77, 52, and 60%, respectively. Amongst all, the AgU showed the highest inhibition percentage. The phytochemicals attached to the nanoparticles and the small size of nanoparticles can be responsible for higher antioxidant activity [59]. Compared to iron oxide nanoparticles, silver nanoparticles showed higher antioxidant activity. The order of antioxidant activity can be given as AgU>AgM>FeU>FeM>fenugreek seed extract. The color effect of DPPH radical scavenging can be seen in Figure 15(b).

## 4. Conclusions

The silver and iron oxide nanoparticles were successfully synthesized by a simple, ecofriendly route using fenugreek seed aqueous extract at room temperature. All nanoparticles were characterized by various techniques to elucidate the stability and functionality of the nanoparticle. The nanoparticles synthesized with the assistance of ultrasound showed higher stability and antibacterial and antioxidant activity due to combined effect of ultrasound and biomolecules adhered on the surface of nanoparticles. The efficacy of fenugreek seed extract as reducing and capping agent was confirmed by FTIR spectra. EDX elemental analysis confirmed that ultrasound enhanced process yield. The morphology of the synthesized nanoparticles was studied using TEM analysis and it revealed that ultrasonication assisted method maintained nearly uniform and relatively small particle size.

## Figures and Tables

**Table 1:** Average crystallite size calculated using the Debye Scherrer equation.

Sample Name	2*θ* (deg)	FWHM (nm)	Plane	Crystallite size (nm)
AgM	38.14	0.501	1 1 1	16.25

AgU	38.16	0.822	1 1 1	9.92

FeM	35.34	1.119	3 1 1	18.60

FeU	35.36	1.167	3 1 1	17.43

## Data Availability

The data used to support the findings of this study are available from the corresponding author upon request.
